# Ten Years Experience With Belatacept-Based Immunosuppression After Kidney Transplantation

**DOI:** 10.14740/jocmr1697w

**Published:** 2014-02-06

**Authors:** Gerrit Grannas, Harald Schrem, Juergen Klempnauer, Frank Lehner

**Affiliations:** a Allgemein-, Viszeral- und Transplantationschirurgie, Medizinische Hochschule, Hannover, Germany

**Keywords:** Immunosuppression, Immunmodulation, Kidney transplantation, Long-term kidney function, Belatacept, Nephrotoxicity, Acute rejection, Chronic allograft Nephropathy

## Abstract

**Background:**

Belatacept was approved for prevention of acute rejection in adult kidney transplantation in 2011 based on two randomized, controlled, multicenter phase 3 studies. Long-term experience over 10 years with belatacept-based immunosuppression after kidney transplantation has not been reported before.

**Patients and Methods:**

Analyzed were 20 patients who had been included into a randomized multicenter phase 2 study by our institution between March 2001 and November 2002. For 10-year follow-up, three different groups could be analyzed: 1) patients with primary calcineurin inhibitor-based (CNI-based) immunosuppression (n = 5), 2) patients with early switch from a belatacept-based to a CNI-based regimen within the first 14 months (n = 8) and 3) patients with completely CNI-free belatacept immunosuppression (n = 7).

**Results:**

Fifteen patients received primary belatacept-based immunosuppression and five patients primary cyclosporine A (CyA). Five patients are still on belatacept. Kidney function measured by serum creatinine levels worsened in the CNI group and the belatacept to CNI switch group during long-term follow-up whereas all patients receiving belatacept throughout follow-up showed stable creatinine values. Acute rejections occurred predominantly in the first 12 months after transplantation and were responsible for four of seven switches from belatacept- to CNI-based immunosuppression within the first 14 months. Five of the 20 patients died.

**Conclusions:**

Belatacept is effective and safe in renal transplant patients and was not associated with graft loss due to chronic allograft nephropathy. Belatacept was well tolerated in all patients and caused less nephrotoxic side effects and was well accepted in most patients.

## Introduction

The early course after kidney transplantation improved considerably since the introduction of calcineurin inhibiting agents for immunosuppression due to reduced rates of acute rejection and graft loss. Novel immunosuppressive agents and combination therapy enabled a reduction of side effects and an increase of graft and patient survival. However, regimens that completely avoid calcineurin inhibitors (CNIs) have been associated with high rates of rejection [1, 2], poorer renal function [3] and intolerability [4, 5]. Nowadays kidney graft loss due to chronic allograft nephropathy (CAN)/interstitial fibrosis and tubular atrophy (IFTA) and death with functioning graft is observed in 3 to 5% of cases after the first year of transplantation [6, 7]. This situation is challenging the transplant community. CAN can accrue from immunologic and non-immunologic impairment, which may be difficult to discriminate in some cases and is still not understood completely. Clinical manifestation might be absent for some time despite of an ongoing pathophysiologic process and is manifested clinically by gradual decrease of renal function frequently accompanied by hypertension and low-grade proteinuria [8]. The main cause of death in patients with a functioning kidney allograft is cardiovascular disease (CVD). Risk factors for CVD are hypertension, hyperlipidemia, diabetes mellitus, obesity and smoking [9]. Hypertension and hyperlipidemia have been shown to be associated with CAN [10, 11]. CNIs have an unfavorable impact on CVD and are known to be nephrotoxic [6, 9]. Underdosing and switching of CNIs may lead to acute rejection and CAN. Therefore, the development of novel immunosuppressive drugs that are equally potent as current drugs with a lower risk of CVD and CAN is still warranted.

Belatacept is a novel immunosuppressive agent, first introduced in kidney transplantation in a phase II trial between March 2001 and December 2003 [12]. Belatacept is a fusion protein composed of the Fc-fragment of a human IgG1 immunoglobulin linked to the extracellular domain of CTLA-4. Mechanism of action is a selective costimulation blockade by binding to the costimulatory ligands CD80/CD86 of antigen-presenting cell surface to inhibit their interaction with the CD28 T-cell receptor. The approval of belatacept is based on two randomized, multicenter, controlled phase 3 studies with start of enrollment in March 2005 and January 2006 respectively [13, 14]. Twelve months’ and three years’ data demonstrated a similar graft and patient survival and superior renal function despite an early increased occurrence of acute rejection and post-transplant lymphoproliferative disorder (PTLD) [13-15]. Moreover, renal pathology by month 12 in this phase II study revealed a lower incidence of CAN among patients receiving belatacept compared to those receiving cyclosporine A (CyA) (29% in the group receiving intensive belatacept and 20% in the group receiving less intensive belatacept versus 44% in the CyA group) with a higher calculated glomerular filtration rate (GFR) in both belatacept groups as compared to the cyclosporine group [12]. Five-year follow-up data demonstrated a stable and still better GFR in patients who were treated with belatacept [16]. Apart from these clinical observations, an immunologic investigation from our institution demonstrated that the soluble CD30 (sCD30) levels that were found in long-term belatacept-treated patients (average treatment 7.8 years) were the same as in healthy controls while the sCD30 concentrations were clearly elevated in the CNI-group [17]. sCD30 is a known predictive serum marker for graft rejection [18, 19] and outcome after kidney transplantation [20]. Furthermore, belatacept regimens were shown to be associated with better cardiovascular and metabolic risk profiles, with lower blood pressure, lower serum lipids and less new onset diabetes mellitus (NODAT) in comparison to CyA at 12 months after transplantation [21]. Thus a lower rate of death with functioning graft and likewise CAN would be expected after kidney transplantation with long-term betalacept-based immunosuppression. Long-term experience over 10 years with belatacept-based immunosuppression after kidney transplantation has not been reported before. Due to termination of long-term follow-up of the phase II trial on October 31, 2012, no centralized data regarding 10-year outcome will be available until long-term follow-up of the registration trials BENEFIT [15] and BENEFIT-EXT [14] in 2015 and 2016 respectively. Furthermore, the outcome of patients who dropped out of the initial phase 2 trial as well as the BENEFIT studies due to rejections, side effects or other events will probably remain unclear. The aim of the present study is to summarize 10 years of experience in our institution with belatacept treatment after kidney transplantation. This is the first systematic analysis including patients who dropped out of the initial phase 2 study due to a switch of immunosuppression.

## Patients and Methods

### Clinical setting

A tertiary referral university hospital within the Eurotransplant area.

### Inclusion and exclusion criteria

From March 2001 to December 2003, a total of 218 patients were enrolled in an open-label, randomized, multicenter, controlled phase 2 study comparing efficiency and safety of belatacept versus CyA (belatacept IM103-100) [15]. Our institution included 20 patients from March 2001 to November 2002. In contrast to the main trial where patients dropped out after immunosuppressive switch from study drugs all of these 20 patients were included into the present analysis. Exclusion criteria were not defined. Thus no essential long-term data of patients who developed immunologic or other problems requiring immunosuppressive switch at any point of time were lost. Because belatacept was developed as an alternative to CNIs for immunosuppression our primary aim was to compare long-term outcome of CNI-treated and CNI-free patients treated with belatacept. The secondary aim was to investigate the outcome of patients who were switched from belatacept to CNI within the first 14 months. As described previously for long-term extension of the primary phase II study and analysis of 5-year follow-up data switches from CyA to tacrolimus (Tac) were allowed [16]. Initial randomization of patients in the “less intensive” or “more intensive” belatacept regimen as well as belatacept dosing during long-term extension (three patients with 8-week dosing and four patients with 4-week dosing) had no influence on the grouping of patients in this retrospective analysis. No significant differences were found in the initial prospective multicenter study after less or more intensive belatacept treatment after 1 year and after 8-week or 4-week dosing in the long-term extension after 5 years [15, 16]. In this retrospective analysis of 10 years long-term data, three different groups could be analyzed: 1) patients with primary CNI-based immunosuppression (n = 5), 2) patients with early switch from a belatacept-based to a CNI-based regimen within the first 14 months after transplantation (n = 8) and 3) patients with completely CNI-free immunosuppression based on belatacept (n = 7).

### Ethical considerations

Analyzed data originate from patients who initially took part in a prospective immunosuppressive trial (belatacept IM103-100). Beside written consent for study participation, all patients were routinely asked for their written consent for anonymous data usage for local research. None of the patients refused data storage, and some patients even consented repeatedly during the 10-year follow-up due to readmission to our hospital. Data analysis and study participation have been approved by our ethics committee (Ethics Commission of Medical School Hannover, Carl-Neuberg-Str. 1, 30625 Hannover, Germany; Head of the Ethics Committee: Prof. Dr. H. D. Troger) prior to the prospective trial and later for additional analysis of the gathered data.

### Clinical data collection

Data were collected retrospectively and prospectively in the context of a larger prospective randomized trial. All patients were routinely examined in our outpatient transplant clinics.

### Study end-points

Study end-points were reasons for immunosuppressive switch, renal function, acute rejection, development of *de novo* malignancies as well as patient and graft survival.

### Operative procedures

Most patients received standard heterotopic kidney transplantation in the iliac fossa from deceased organ donation (DOD) or living donation. One patient received a transabdominal simultaneous unilateral nephrectomy with subsequent orthotopic kidney transplantation, one patient received a simultaneous infrarenal aorto-bifemoral prosthesis with subsequent transplantation of the kidney with anastomosis of the renal artery on the aorto-bifemoral bypass and the third patient needed an additional thrombenarteriectomy of the common iliac artery.

### Immunosuppression

Patients were initially treated according to study design as described before [15]. Switch from belatacept to CNI (either CyA or tacrolimus) resulted instead of drop out into the third observational “switch group”.

### Antifungal, antibiotic and antiviral prophylaxis

Standard prophylaxes consisted of 800/160 mg trimethoprim/sulfamethoxazole (Cotrim forte^®^, Hexal) administered three times weekly for 6 months and topical amphotericine B (Ampho-Moronal^®^, Bristol-Myers Squibb) in the postoperative period. Additionally, all patients with a major risk profile for cytomegalovirus (CMV) (donor CMV IgG +/recipient CMV IgG -) received gancyclovir (Cymeven^®^, Roche) i.v. during the early postoperative phase followed by oral administration of valganciclovir (Valcyte^®^, Roche) for a minimum of 3 months adjusted to kidney function. These prophylactic regimens were adopted from the local routine practice at the time of kidney transplantation at the Hannover Medical School.

### Follow-up

All patients were regularly seen in the outpatient transplant clinics. Follow-up included routine laboratory tests, regular determination of renal function and abdominal ultrasound evaluations. Furthermore, patients were included into a routine biopsy program with allograft biopsies at baseline after reperfusion or during back table preparation of the graft and during follow-up at 3, 6 and 12 months after transplantation. A diagnosis of rejection of the kidney allograft was based on more than 25% elevation of serum creatinine from baseline values with histologic confirmation of rejection or histologic confirmation alone in case of typical findings in a routine biopsy (silent rejection) in all cases.

During 10-year follow-up, different methods of GFR measurement were used. In our institution, the method changed from classic GFR measurement by using 24-h urin collection to a cystatin C-estimated method. Affiliated nephrologic practices used eGFR based on MDRD/abbreviated MDRD or Cockroft and Gault estimation. Therefore, we waived assessment and comparison of kidney function by GFR and considered serum-creatinine for follow-up of graft function.

### Statistics

Mann-Whitney U test and log-rank tests were applied where appropriate. For all statistical tests, the level of significance was defined as P < 0.05. The SPSS statistics software version 20.0 (IBM, Somers, NY, USA) was used to perform statistical analysis.

## Results

### Patients

Between March 2001 and November 2002, 20 kidney transplantation recipients were included at Hannover Medical School and were randomized into a phase 2 study comparing different regimens of belatacept versus CyA. The median age was 41 years ranging from 25 to 70 years. Table 1 provides a detailed overview of the underlying diagnoses and the most pertinent findings.

**Table 1:** Data of Patients Primarily Enrolled Into the Phase II Trial With and Developed Third Observational Group Due to Switch From Belatacept-Based to CNI-Based Immunosuppression (Switched Patients)

Pat. No.	Age (years)	Gender	BMI (kg/m^2^)	No. of CRF	Tye of organ	Donor age (years)	CIT (min)	MM	Underlaying disease
Patients initially receiving belatacept (*switched patients)									
1*	53	M	29.8	3	LRD	57	222	2	Nephrosklerosis
2	44	M	19.4	3	DOD	23	844	2	Unknown
3*	46	M	23.6	1	LNRD	45	226	5	Chronic glomerulonephritis
4*	32	M	23.1	1	DOD	58	980	0	IgA nephropathy
5*	41	M	21	2	DOD	46	1,273	1	IgA nephropathy
6	53	M	25.1	3	DOD	38	849	2	Polycystic kidney disease
7	40	M	30	2	LRD	64	205	3	IgA nephropathy
8	27	M	18.9	1	DOD	37	717	2	Obstructive nephropathy
9*	45	M	27.5	2	DOD	29	1,430	2	Nephrosklerosis
10	41	M	19.5	2	DOD	58	821	4	Unknown
11*	25	M	25.6	2	DOD	58	559	0	IgA nephropathy
12*	70	M	24.2	2	DOD	36	820	1	Nephrosklerosis
13	32	M	22.3	3	DOD	53	1,233	3	Obstructive nephropathy
14*	42	F	20.7	2	DOD	58	856	0	Post-streptococcal glomerulonephritis
15	32	F	18.1	2	DOD	54	786	0	Hereditary nephritis
Patients initially receiving CyA									
16	60	M	22.4	2	DOD	17	841	1	Chronic pyelonephritis
17	36	M	22.7	1	DOD	49	784	2	Chronic glomerulonephritis
18	24	M	20.2	1	DOD	48	1,179	3	Cystinosis
19	41	M	23.5	2	DOD	58	1,118	0	IgA nephropathy
20	58	F	27.8	1	DOD	38	1,154	2	Chronic pyelonephritis

BMI: body mass index; CRF: cardiovascular risk factors; CIT: cold ischemia time; MM: mismatch. *: switched patients.

Five patients suffered from IgA nephropathy, three from nephrosclerosis, two in each case from chronic glomerulonephritis, obstructive nephropathy and chronic pyelonephritis and one from cystinosis, hereditary nephritis, poststreptococcal nephritis and adult polycystic kidney disease each. In two patients underlying disease was unknown. Median recipient body mass index (BMI) was 22.9, range: 18.1-30.0. Prior to transplantation patients had median two (range 1-3) of six established cardiovascular risk factors (obesity, lipid/cholsetrol, tobacco, diabetes, alcohol and hypertension). Three patients received an organ from living donors, two as a living related donation (LRD) and one as a living nonrelated donation (LNRD). The other recipients received a transplant from deceased donors.

#### Donors

The organs were accepted on the basis of standard criteria. Only ABO-compatible donors with no evidence of malignancy or hepatitis B or C infection were accepted. Median donor age was 49 years ranging from 17 to 64 years.

### Development of observational groups

**Figure 1:**
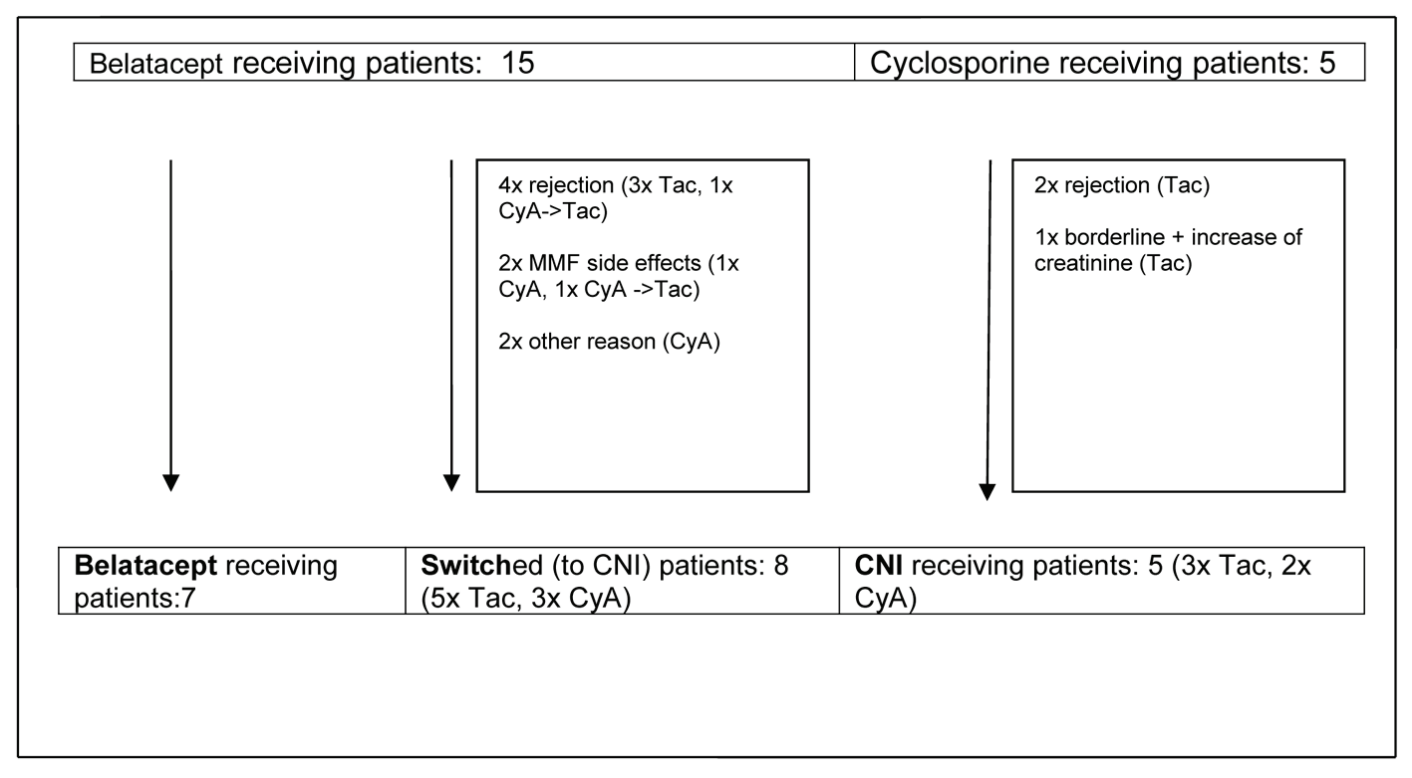
Development of observational groups up to month 14 post-transplant. CyA: cyclosporine A; Tac: tacrolimus; CNI: calcineurin inhibitor; MMF: mycophenolate mofetil.

During the first 14 months rejections, side effects and other events led to reconfiguration of initially two main observational groups - primary CNI-treated and CNI-free patients - into three groups containing a group of patients switched from belatacept to CNI (Fig. 1).

#### Rejections

Three patients were switched after steroid pulse therapy from belatacept to CNI (2× Tac, 1× CyA) due to clinically suspected and biopsy proven rejection (months 1, 2 and 6) and one due to recurrent subclinical rejection detected by routine biopsy at months 3 and 6. The first rejection episode was treated with steroid pulse therapy, the second episode with switch to Tac.

Another belatacept receiving patient with a subclinical rejection episode at month 3 was successfully treated with steroid pulse therapy.

One patient of the CyA group developed clinically suspected and biopsy proven rejection at month 3 treated with steroids and switch to Tac.

One patient who was already switched from belatacept to CyA due to MMF-induced agranulocytosis, developed clinically suspected and biopsy proven rejection at month 8 treated with steroids and switch to Tac.

Furthermore, three borderline changes were detected by routine biopsy. Two of them in the CyA group at months 3 and 6 were treated because of a slight increase of serum creatinine (around 15% from baseline values). One patient received 250 mg methylprednisolone i.v. daily for 3 consecutive days, the other was additionally switched from CyA to Tac because MMF was paused at this time after severe and lengthened CMV infection during month 2. In both patients, a complete return to baseline creatinine level could be achieved. The patient with borderline changes in the belatacept group was not treated because of an unimpaired serum creatinine and stable creatinine clearance. Follow-up biopsy at month 6 showed no signs of immunological activity.

#### Side effects

Two belatacept receiving patients suffered from side effects leading to immunosuppressive switch. One developed agranolocytosis at month 6 which was presumably MMF associated. In the course of MMF discontinuation this patient was additionally switched to CyA according to the study design of the initial phase II study. The other patient developed enduring diarrhea and was switched at month 14 after discontinuation of MMF to CyA, too.

#### Other events

One initial belatacept receiving patient was switched to CyA due to refusal of inform consent of the initial phase II study on day 1 and one patient later during follow-up in the course of surgical treatment of gastic perforation at month 1.

### Infections

Bacterial infections were only seen in three patients in the belatacept group. Two patients developed an urosepsis at months 3 and 6 after transplantation. The patient who suffered from urosepsis at month 6 was already switched to CyA 6 days after transplantation because of withdrawn consent. The third patient had a complicated course following aortal and mitral valve replacement 6 months after transplantation followed by endocarditis leading to a second valve replacement 11 months after Tx and subsequently recurrent pleural empyema that finally resulted in septic death 17 months after Tx.

CMV infections occurred overall in seven patients (Table 2). Most CMV infections were found after discontinuation of CMV prophylaxis in patients with a high risk profile (donor CMV IgG +/recipient CMV IgG -). One patient suffered from three episodes of CMV infections and gancyclovir resistance requiring at foscarnet (Foscavir, AstraZeneca) treatment. Between the first and second infection episode, this patient developed an acute rejection after reduction of MMF doses. This patient was consequently treated with steroid bolus therapy and switch of immunosuppression from belatacept to tacrolimus. The other patient (donor CMV IgG +/recipient CMV IgG +) with two infection episodes and patients with only one infection episode were successfully treated with intravenous gancyclovir and temporary reduction of MMF in each case.

**Table 2:** CMV IgG Status and Post-Transplant Infections/Malignancies

Pat. No.	CMV donor	CMV recipient	Viral infection	Bacterial infection	malignancies
Patients initial treated with belatacept					
2	pos	pos	-	-	-
6	neg	pos	-	-	2× basalioma (year 5)
					Bronchial carcinoma (year 8)
7	pos	neg	CMV (month 5)	-	-
8	neg	neg	-	-	-
10	neg	pos	Herpes zoster (month 2)	-	-
13	neg	pos	-	Endocarditis, pleural empyema	-
15	pos	neg	CMV (month 8)	Urosepsis (month 6)	CIN cervix (year 7)
Patients switched from belatacept to CNI					
1	pos	neg	3× CMV (month 5, 6, 7)	-	-
3	pos	neg	Herpes zoster (year 2)	-	-
4	neg	neg	-	-	-
5	neg	pos	-	-	-
9	neg	pos	-	-	-
11	neg	pos	-	-	-
12	pos	pos	CMV (month 1, 3)	Urosepsis (month 3)	Bronchial carcinoma (year 2)
14	pos	pos	-	-	-
CNI-treated patients					
16	pos	neg	-	-	Basalioma (year 8)
17	pos	neg	CMV (month 6)	Urosepsis (year 6)-	-
18	pos	neg	CMV (month 2)	-	-
19	pos	neg	CMV (month 4)	-	2× basalioma (year 6, 9)
20	pos	pos	-	Pseudomembraneous colitis (month 2)	-

CMV: cytomegalovirus; CIN: cervical intraepithelial neoplasia; CNI: calcineurin inhibitor.

### Long-term follow-up

Remarkably, as described above nearly all switches of immunosuppression (n = 7) in the belatacept group occurred during the first year after transplantation. Only one patient was switched shortly later (month 14), and all other patients (n = 7) remained on belatacept as base immunosuppressive therapy. Finally three different groups of patients could be analyzed: 1) patients who were receiving a primary CNI-based immunosuppressive regimen, 2) patients with early switch to a CNI-based regimen and 3) patients who stayed on a CNI-free immunosuppressive regimen with belatacept (Fig. 1, Table 3).

**Table 3:** 10-Year Follow-Up of Serum-Creatinine and Immunosuppression

Pat. No.		14	2 yrs	3 yrs	4 yrs	5 yrs	6 yrs	7 yrs	8 yrs	9 yrs	10 yrs
		mon									
CNI-free patients treated with belatacept (1)											
2	Immunosup	Bela	Bela	Bela	Bela	Bela	Bela	Bela	Bela	Bela	Bela
	Creatinine, μmol/L	70	71	73	66	77	72	66	68	76	76
6		Bela	Bela	Bela	Bela	Bela	Bela	Bela	Bela	Patient died	
		86	86	96	91	-	82	-	75		
7		Bela	Bela	Bela	Bela	Bela	Bela	Bela	Bela	Bela	Bela
		146	143	151	132	-	139	-	-	-	128
8		Bela	Bela	Bela	Bela	Bela	Bela	Bela	Bela	Bela	Bela
		117	101	113	107	94	109	95	90	98	97
10		Bela	Bela	Bela	Bela	Bela	Bela	Bela	Bela	Bela	Bela
		113	112	116	116	134	118	108	108	115	118
13		Bela	Patient died								
		156									
15		Bela	Bela	Bela	Bela	Bela	Bela	Bela	Bela	Bela	Bela
		76	93	89	103	112	98	92	98	99	91
Median		113	103	105	105	103	104	94	90	99	97
Patients switched from belatacept to CNI (2)											
1		TAC	TAC	TAC	TAC	TAC	TAC	TAC	TAC	TAC	TAC
		134	149	149	131	149	165	182	147	181	194
3		TAC	TAC	TAC	TAC	TAC	TAC	TAC	TAC	TAC	TAC
		237	251	250	202	219	182	214	196	194	219
4		TAC	TAC	TAC	TAC	TAC	TAC	TAC	TAC	TAC	TAC
		189	184	220	180	189	207	206	199	211	197
5		CyA	CyA	CyA	CyA	CyA	CyA	CyA	CyA	CyA	CyA
		122	118	126	121	127	116	117	107	102	116
9		Bela	Switch month 14 to CyA; patient died at month 20								
		115									
11		TAC	TAC	TAC	TAC	TAC	TAC	TAC	TAC	TAC	Tx failure
		149	149	127	182	190	192	262	379	674	
12		CyA	CyA	Patient died							
		180	161								
14		TAC	TAC	TAC	TAC	TAC	TAC	TAC	TAC	TAC	TAC
		118	130	119	130	110	160	129	125	143	123
Median		142	149	138	156	169	174	194	172	188	194
CNI-treated patients (3)											
16		TAC	TAC	TAC	TAC	TAC	TAC	TAC	TAC	Patient died	
		201	250	219	273	266	274	313	362		
17		CyA	TAC	TAC	TAC	TAC	TAC	TAC	TAC	TAC	TAC
		200	207	182	199	195	189	179	196	207	194
18		TAC	TAC	TAC	TAC	TAC	+Aza	+Aza	+Aza	+Aza	+Aza
		130	170	180	183	237	266	248	189	179	179
19		CyA	CyA	CyA	CyA	CyA	CyA	CyA	CyA	CyA	CyA
		95	138	127	129	113	124	108	122	113	120
20		CyA	CyA	CyA	CyA	CyA	+Aza	+Aza	+Aza	+Aza	+Aza
		95	107	104	106	126	118	106	102	102	109
Median		130	170	180	183	195	189	179	189	146	150
Mann-Whitney U test; P	1 vs. 2	0.04	0.005	0.026	0.015	0.067	0.009	0.009	0.009	0.019	0.056
	1 vs. 3	0.268	0.03	0.052	0.052	0.063	0.017	0.032	0.016	0.114	0.111
	1 vs. 2+3	0.046	0.002	0.01	0.007	0.026	0.002	0.003	0.002	0.014	0.029

Bela: belatacept; TAC: tacrolimus; CyA: ciclosporine A; Aza: azathioprine.

#### Cardiovascular events

During follow-up cardiovascular events occurred in 2/5 patients (40%) in the CNI group, 5/8 patients (63%) in the switch group and 1/7 patients (14%) in the belatacept group. Three patients developed two cardiovascular events (n = 2 in the switch group and n = 1 in the belatacept group). The patient in the belatacept group suffered from pretransplant mitral and aortal valve insufficiency in combination with coronary stenosis requiring mitral and aortal valve replacement in combination with coronary bypass at month 6 post Tx and re-replacement of valves at month 12 due to postoperative endocarditis. After the second operation this patient developed recurring pleural empyema leading to death by septic organ failure as described before. Of the patients in the switch group, one needed percutaneous transluminal angioplasty (PTA) and stenting of the right external iliac artery at month 11 after Tx and the left and right common iliac arteries at year 4 due to progress of preexisting peripheral artery occlusive disease (PAOD). The other patient needed aortic replacement operation due to an infrarenal aortic aneurism at year 3 after Tx and developed a stroke of the arteria cerebri media at year 8.

From the other five patients, who developed only one cardiovascular event, two patients died: one in the switch group due to sudden heart death and one in the CNI group due to decompensation of aortic valve stenosis. Further events are given in Table 4.

**Table 4:** Cardiovascular Events

Pat. No.	Group	Cardiovascular event
13	Belatacept	Mitral and aortal valve insufficiency (month 4 and 12)
1	Switch	Abdominal aortic aneurism (year 3); stroke (arteria cerebri media; year 8)
5	Switch	Stenosis of right external iliac artery (month 11);
		Stenosis of left and right common iliac artery (year 4)
9	Switch	Sudden heart death (month 20)
11	Switch	Stenosis of right coronary artery (year 7)
14	Switch	Stenosis of transplant artery (month 7)
16	CNI	Aortic valve stenosis with decompensation (year 9)
18	CNI	Stroke (arteria cerebri media; year 4)

#### Cardiovascular risk factors

For the control of blood pressure at month 14 post-transplantation, a median intake of three drugs was necessary in the belatacept and switch groups whereas patients in the CNI group already needed a combination of four antihypertensive drugs. The median number of antihypertensive drugs decreased during follow-up to two in the belatacept group whereas patients in the switch group and in the CNI group required a median of four antihypertensive drugs for blood pressure control after 10 years. The percentage of patients with lipid lowering medication increased only in the switch group.The only case of an NODAT was observed in the switch group (Table 5).

**Table 5:** Cardiovascular Risk Factors and Medical Treatment

Parameter	14 mon	3 yrs	5 yrs	8 yrs	10 yrs
Systolic BP median					
Belatacept	120	130	132	122	120
Switch	126	123	130	125	120
CNI	135	126	128	139	122
Diastolic BP (mmHg; median)					
Belatacept	74	75	77	75	68
Switch	79	80	80	79	80
CNI	82	76	85	83	74
No. of BPM (median; (range))					
Belatacept	3 (2-5)	3 (2-5)	3 (1-5)	2 (1-5)	2 (1-4)
Switch	3 (2-4)	3 (2-6)	4 (2-6)	4 (2-4)	4 (2-4)
CNI	4 (2-4)	3 (3-4)	3 (2-4)	3 (3-4)	4 (3-5)
Cholesterol (mg/dL; median)/triglycerides					
Belatacept	193/171	233/165	186/112	226/143	232/118
Switch	174/343	195/508	162/259	176/238	197/334
CNI	209/175	186/187	205/188	255/145	231/166
Pat. with lipid lowering medication					
Belatacept	4 (57%)	3 (50%)	4 (67%)	4 (67%)	3 (60%)
Switch	4 (50%)	4 (67%)	5 (83%)	4 (67%)	4 (80%)
CNI	2 (40%)	3 (60%)	2 (40%)	2 (40%)	1 (25%)
NODAT (n (%))					
Belatacept	0	0	0	0	0
Switch	1 (14%)	1 (17%)	1	1	1 (20%)
CNI	0	0	0	0	0
Number of patients					
Belatacept	7	6	6	6	5
Switch	8	6	6	6	5
CNI	5	5	5	5	4

BP: blood pressure; mon: months.

#### Kidney function

**Figure 2:**
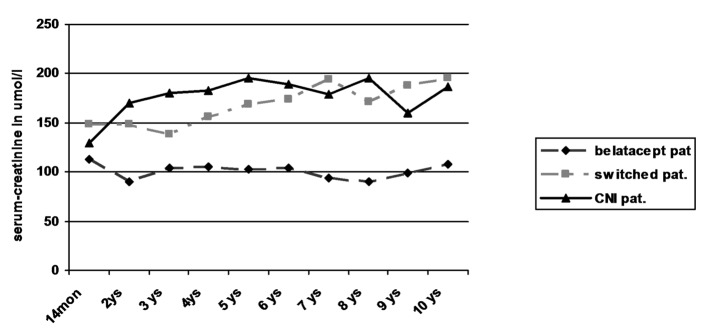
Kidney function by serum-creatinine.

Regarding creatinine values in the three patient groups, only the patients still receiving belatacept showed a stable kidney function without any graft loss due to graft failure (Table 3; Fig. 2). In contrast, two of seven switched patients (29%) showed an increase of creatinine by more than 50% from baseline values over time with graft failure in one of the patients. Kidney biopsy in one patient with graft failure at the time of declining function revealed recurrence of IgA nephropathy in combination with signs of chronic CNI toxicity. Among the patients receiving primary CNI, two of five patients (40%) developed an increase of creatinine of more than 50% from baseline values. Due to worsening of kidney function in one of the patients graft biopsy was performed 6 years after transplantation which revealed evidence of chronic CNI toxicity accompanied with 20% tubular atrophy of 20% and minimal nephrocalcinosis. Both patients were treated with minimization of CNI in combination with the use of azathioprine leading to stabilization of graft function in one and even slight amelioration of creatinine values in the other patient. No graft failure occurred during observational period.

Despite the low number of patients in each group statistical comparison of the belatacept group with the switch and CNI group revealed significantly lower creatinine levels in the belatacept group starting after the first year (P < 0.05; Mann-Whitney U test).

#### Malignancies

Overall five patients developed malignancies (Table 2). In the belatacept group, one patient (44 years old at study start, male, around 30 package years) developed skin cancer (basalioma, 5 years after transplantation) and bronchial cancer 8 years after transplantation and died 6 months later. The other patient (38 years old at study start, female) developed cervical intraepithelial neoplasia (CIN) 7 years after TX. In the switch group, one patient (70 years old at study start, male, more than 50 package years) developed a bronchial carcinoma 3 years after transplantation and died after 4 months. In the CyA group, two patients developed skin cancer (basalioma): one patient (41 years old at study start, male) at years 6 and 9 after Tx, the other patient (60 years old at study start, male) at year 8 after Tx.

#### Patient and graft survival

During the 10-year observational period, five of 20 patients died (two in the belatacept group, two in the switch group and one in the CyA group). One patient developed CAN and received a preemptive living related kidney retransplantation (ABO incompatible) 10 years after primary transplantation. Two patients died as a result of bronchial cancer. One patient died from infectious complications following valve replacement as described above. One patient died due to decompensation of aortal valve stenosis and one due to sudden heart failure (Table 6).

**Table 6:** Patient and Graft Survival

Belatacept group	Deaths: 2	- Sepsis due to pleural empyema after valve replacement (year 2)
		- Bronchial cancer (year 8)
	Graft loss: 0	
Switch group	Deaths: 2	- Sudden heart failure (month 20)
		- Bronchial cancer (year 3)
	Graft loss: 1	-Recurrence of IgA nephropathy (year 9)
CNI group	Deaths: 1	- Decompensation of aortic valve stenosis (year 9)
	Graft loss: 0	

## Discussion

Due to approval of belatacept (Nulojix) as immunosuppressant agent in kidney transplantation, the long-term observational extension of the phase II study was already terminated. So far published data on the long-term use of this drug are limited to 5 years data of the phase II trial [16] and 3 years data from the BENEFIT and BENEFIT EXT trials [14, 22]. Nowadays the purpose in kidney transplantation is to extend graft survival beyond 10 years and to reduce adverse effects of immunosuppressive treatment. Accordingly 10 years experience in usage of belatacept is of peculiar interest.

Our patients tolerated this treatment well despite of intravenous application more frequent visits to the outpatient clinic over many years. No patient had to be switched to an oral immunosuppressive regimen because of refusal of intravenous application. One patient withdrew consent to the study and therefore to further belatacept administration immediately after transplantation beset by doubts concerning novel immunosuppressive drugs. Despite mandatory intravenous application of belatacept which appears to be a significant disadvantage in maintenance treatment at first sight we find that this disadvantage also offers advantages in patients who tend to be non-compliant, have problems with oral intake, pharmacologic interactions or enteral malabsorption. Especially non-compliance, which is responsible for up to 16% of graft losses [23, 24] and an increase of health care costs by approximately $33,000 3 years after Tx [25], might be reduced by intravenous therapy and steady visits.

Rejections were the most common reason for switching patients from belatacept to CNI. Until reconfiguration of the initial phase II patient groups into our observational patient groups at month 14 post-transplantation more rejection episodes were observed in patients initially treated with belatacept: 50% (7/14; patient refused inform consent on day 1 was excluded) vs. 20% (1/5) in the CyA group. Except of one rejection all episodes occurred during the first 6 months after transplantation, which lead to a rate of 43% at 6 month in the belatacept group. Regarding the published data of the multicenter phase II study at this point of time a rejection rate (clinically suspected and biopsy proven + treated subclinical rejection) of 15 and 21% in the belatacept groups and 15% in the CyA group [12] was given, so that the difference in our rate of rejections seemed to be a local singularity. Severity of rejections ranged from Banff Ia to IIa. Due to lack of experience with the new immunosuppressant only subclinical rejections were treated with steroids alone, rejections with decrease of kidney function were additionally switched to CyA or Tac. After remodeling of the initial phase II study groups at post-transplant month 14 late rejections were rare within 10 years. Only one patient in the belatacept group developed an acute rejection episode after discontinuation of MMF due to pleural empyema and sepsis.

In the switch group half of the patients suffered from acute rejection during the first 14 months after transplantation. Considering that acute rejections showed a negative impact on long-term graft survival [26], it could be assumed that the switch group would have the worst results of long-term outcome. But regarding our investigation of dropped out (switched) patients a negative impact was not obvious. As expected kidney function based on serum creatinine levels was indeed worse in comparison with the belatacept group but very similar to the CNI group. These results match nicely with the results of the BENEFIT and BENEFIT-EXT trials which showed that nearly all acute rejection episodes occurred during the first 6 months after transplantation [13, 14] and demonstrated that the GFRs at month 12 were higher in belatacept patients [13, 14] despite of a higher incidence of acute rejections [7] and more grade IIb rejections [13, 14]. These results allowed the presumption that rejection episodes in patients receiving belatacept are less problematic in view of long-term graft function and survival in comparison to rejections in patients receiving CNI. Among others a lesser development of donor-specific anti-HLA antibodies after rejection episodes might contribute to this [13, 15].

Despite the highest number of patients (n = 3) with more than two pretransplant cardiovascular risk factors only one of seven patients (14%) in the belatacept group had a cardiovascular event (Table 1). In the CNI group, 2/5 patients (40%) and in the switch group 5/8 patients (63%) suffered from cardiovascular events. The median number of antihypertensive drugs could be reduced to two during the 10-year follow-up only in the belatacept group. In the CNI and switch groups, the median number of antihypertensive drugs remained constant or even increased to a median of four. This superior outcome of patients treated with belatacept might be a beneficial effect of CNI avoidance that nevertheless had no observable influence on graft and patient survival in our patients probably due to the low number of patients that were examined.

Overall survival of patients was 100% at 1 year, 85% after 5 years and 75% after 10 years. There was no obvious difference in the three groups. Two patients each died in the belatacept group and switch group, one patient in the CNI group. Graft survival was mainly terminated by patient death with functioning graft. Only one graft loss was observed in the switch group due to recurrence of IgA nephropathy without any influence on patient’s survival. However kidney function on the basis of serum creatinine worsened significantly in the CNI and switch groups during long-term follow-up when compared to patients receiving belatacept who showed stable creatinine values since transplantation (Table 3, Fig. 2). The 3-year data of the BENEFIT trial found similar results. This difference even increased during the 3-year period from 15 mL/min/1.73 m^2^ at year 1 to 21 mL/min/1.73 m^2^ at year 3 [15]. No differences in the development of graft function could be seen in the comparison of the switch with the CNI group despite the fact that four of the eight switched patients had early rejections. Interestingly in two patients in the CNI group with increasing creatinine azathioprine was added with minimization of CNI during post-transplant year 6: one due to typical signs of chronic CNI toxicity in graft biopsy, the other due to suspected CNI toxicity. Recent immunologic investigations of patients treated with belatacept revealed different compositions of T-cell subpopulations with less IL-17 production in comparison to patients receiving CNI [17]. This and the observations of this current study as well as the previously published results [15, 16, 22] suggest that long-term graft function may be significantly superior in patients with belatacept-based long-term immunosuppression.

In conclusion, this report demonstrates the feasibility of long-term immunosuppression with belatacept in kidney transplantation. Frequent i.v. administration was well tolerated and moreover resulted in good adherence. Belatacept seems to be effective with advantages in cardiovascular risk profiles and seems to lead to superior long-term kidney function. Patients who needed to be switched from belatacept to CNI do not seem to have any disadvantage in comparison to primary CNI-treated patients but seem to lose the advantages of long-term belatacept treatment as shown by the current results.
